# Supplementary material for: Effects of maternal anemia on low-birth-weight in Sub-Sahara African countries: Systematic review and meta-analysis
Source: PLoS One. 2025 Jun 25;20(6):e0325450. doi: 10.1371/journal.pone.0325450 (PMC12192055; doi:10.1371/journal.pone.0325450)
Supplement: S1 Table — (DOCX) [file pone.0325450.s001.docx]

| **Pubmed data base Final Searching Date: June 12, 2024** | | | | |
| --- | --- | --- | --- | --- |
| **Concept** | **Line** | **Search term** | **Search strategy** | **Records** |
| Anemia | #1 | Anemia during pregnancy, Maternal anemia, anemia, Maternal anaemia, anaemia, Hemoglobin, maternal hemoglobin, Haemoglobin, Maternal Haemoglobin, hematocrit, Maternal hematocrit, Haematocrit,  Maternal haematocrit, Maternal hemoglobin level,  Maternal hemoglobin concentration | "Anemia during pregnancy"[Title/Abstract] OR "Maternal anemia"[Title/Abstract] OR "Anemia"[Title/Abstract] OR "Maternal anaemia"[Title/Abstract] OR "anaemia"[Title/Abstract] OR "Hemoglobin"[Title/Abstract] OR "maternal hemoglobin"[Title/Abstract] OR "Haemoglobin"[Title/Abstract] OR "Maternal Haemoglobin"[Title/Abstract] OR "Hematocrit"[Title/Abstract] OR "Maternal hematocrit"[Title/Abstract] OR "Haematocrit"[Title/Abstract] OR "Maternal haematocrit"[Title/Abstract] OR "Maternal hemoglobin level"[Title/Abstract] OR “Maternal hemoglobin concentration” [Title/Abstract] OR "Anemia"[MeSH Terms] OR "anemia, iron deficiency"[MeSH Terms] OR "Hemoglobins"[MeSH Terms] OR "Hematocrit"[MeSH Terms] | 498,888 |
| Low birth weight | #2 | Low birth weight,  Adverse birth outcome, Perinatal outcome,  Birth outcome,  Birth weight,  Low for gestational age | "Low birth weight"[Title/Abstract] OR "Adverse birth outcome"[Title/Abstract] OR "Perinatal outcome"[Title/Abstract] OR "Birth outcome"[Title/Abstract] OR "birth weight"[Title/Abstract] OR "low for gestational age"[Title/Abstract] OR "infant, low birth weight"[MeSH Terms] OR "infant, extremely low birth weight"[MeSH Terms] OR "infant, very low birth weight"[MeSH Terms] OR "birth weight"[MeSH Terms] | 118,059 |
| Sub-Saharan African Countries | #3 | Angola, Benin, Botswana,  Burkina Faso, Burundi, Cape Verde, Cameroon, Central African Republic, Chad, Comoros, Democratic Republic of the Congo, Cote D'ivoire, Equatorial Guinea,  Eritrea, Eswatini, Ethiopia, Gabon, Gambia, Ghana, Guinea, Guinea-Bissau, Kenya, Lesotho, Liberia, Madagascar, Malawi, Mali, Mauritania, Mauritius, Mozambique, Namibia, Niger, Nigeria, Rwanda, Sao Tome and Principe, Senegal, Seychelles, Sierra Leone, Somalia, South Africa, South Sudan, Sudan, Tanzania, Togo, Uganda, Zambia, Zimbabwe, africa south of the sahara, Sub-Saharan africa, subsaharan africa, central africa, north africa, northern africa, sahara, southern africa, east africa, eastern africa, West africa, western africa | "Angola"[MeSH Terms] OR "Benin"[MeSH Terms] OR "Botswana"[MeSH Terms] OR "Burkina Faso"[MeSH Terms] OR "Burundi"[MeSH Terms] OR "Cabo Verde"[MeSH Terms] OR "Cameroon"[MeSH Terms] OR "Central African Republic"[MeSH Terms] OR "Chad"[MeSH Terms] OR "Comoros"[MeSH Terms] OR "Democratic Republic of the Congo"[MeSH Terms] OR "cote d ivoire"[MeSH Terms] OR "Equatorial Guinea"[MeSH Terms] OR "Eritrea"[MeSH Terms] OR "Eswatini"[MeSH Terms] OR "Ethiopia"[MeSH Terms] OR "Gabon"[MeSH Terms] OR "Gambia"[MeSH Terms] OR "Ghana"[MeSH Terms] OR "Guinea"[MeSH Terms] OR "Guinea-Bissau"[MeSH Terms] OR "Kenya"[MeSH Terms] OR "Lesotho"[MeSH Terms] OR "Liberia"[MeSH Terms] OR "Madagascar"[MeSH Terms] OR "Malawi"[MeSH Terms] OR "Mali"[MeSH Terms] OR "Mauritania"[MeSH Terms] OR "Mauritius"[MeSH Terms] OR "Mozambique"[MeSH Terms] OR "Namibia"[MeSH Terms] OR "Niger"[MeSH Terms] OR "Nigeria"[MeSH Terms] OR "Rwanda"[MeSH Terms] OR "Sao Tome and Principe"[MeSH Terms] OR "Senegal"[MeSH Terms] OR "Seychelles"[MeSH Terms] OR "Sierra Leone"[MeSH Terms] OR "Somalia"[MeSH Terms] OR "South Africa"[MeSH Terms] OR "South Sudan"[MeSH Terms] OR "Sudan"[MeSH Terms] OR "Tanzania"[MeSH Terms] OR "Togo"[MeSH Terms] OR "Uganda"[MeSH Terms] OR "Zambia"[MeSH Terms] OR "Zimbabwe"[MeSH Terms] OR "africa south of the sahara"[MeSH Terms:noexp] OR "africa, central"[MeSH Terms:noexp] OR "africa, northern"[MeSH Terms:noexp] OR "africa, southern"[MeSH Terms:noexp] OR "africa, eastern"[MeSH Terms:noexp] OR "africa, western"[MeSH Terms:noexp] OR ((("Angola"[Title/Abstract] OR "Angolan"[Title/Abstract] OR "Benin"[Title/Abstract] OR "Beninese"[Title/Abstract] OR "Botswana"[Title/Abstract] OR "Botswanan"[Title/Abstract] OR "Burkina Faso"[Title/Abstract] OR "Burkinabe"[Title/Abstract] OR "Burundi"[Title/Abstract] OR "Burundian"[Title/Abstract] OR "Cape Verde"[Title/Abstract] OR "Cape Verdean"[Title/Abstract] OR "Cameroon"[Title/Abstract] OR "Cameroonian"[Title/Abstract] OR "Central African Republic"[Title/Abstract] OR "Central African"[Title/Abstract] OR "Chad"[Title/Abstract] OR "Chadian"[Title/Abstract] OR "Comoros"[Title/Abstract] OR "Comorian"[Title/Abstract] OR "Democratic Republic of the Congo"[Title/Abstract] OR "Congolese"[Title/Abstract] OR "cote d ivoire"[Title/Abstract] OR "Ivorian"[Title/Abstract] OR "Equatorial Guinea"[Title/Abstract] OR "Equatorial Guinean"[Title/Abstract] OR "Eritrea"[Title/Abstract] OR "Eritrean"[Title/Abstract] OR "Eswatini"[Title/Abstract] OR "Swazi"[Title/Abstract] OR "Ethiopia"[Title/Abstract] OR "Ethiopian"[Title/Abstract] OR "Gabon"[Title/Abstract] OR "Gabonese"[Title/Abstract] OR "Gambia"[Title/Abstract] OR "Gambian"[Title/Abstract] OR "Ghana"[Title/Abstract] OR "Ghanaian"[Title/Abstract] OR "Guinea"[Title/Abstract] OR "Guinean"[Title/Abstract] OR "Guinea-Bissau"[Title/Abstract) OR "Guinea-Bissauan"[Title/Abstract]) OR "Kenya"[Title/Abstract] OR "Kenyan"[Title/Abstract] OR "Lesotho"[Title/Abstract] OR "Mosotho"[Title/Abstract] OR "Liberia"[Title/Abstract] OR "Liberian"[Title/Abstract] OR "Madagascar"[Title/Abstract] OR "Malagasy"[Title/Abstract] OR "Malawi"[Title/Abstract] OR "Malawian"[Title/Abstract] OR "Mali"[Title/Abstract] OR "Malian"[Title/Abstract] OR "Mauritania"[Title/Abstract] OR "Mauritanian"[Title/Abstract] OR "Mauritius"[Title/Abstract] OR "Mauritian"[Title/Abstract] OR "Mozambique"[Title/Abstract] OR "Mozambican"[Title/Abstract] OR "Namibia"[Title/Abstract] OR "Namibian"[Title/Abstract] OR "Niger"[Title/Abstract] OR "Nigerien"[Title/Abstract] OR "Nigeria"[Title/Abstract] OR "Nigerian"[Title/Abstract] OR "Rwanda"[Title/Abstract] OR "Rwandan"[Title/Abstract] OR "Sao Tome and Principe"[Title/Abstract] OR "Sao Tomeans"[Title/Abstract] OR "Santomeans"[Title/Abstract] OR "Senegal"[Title/Abstract] OR "Senegalese"[Title/Abstract] OR "Seychelles"[Title/Abstract] OR "Seychellois"[Title/Abstract] OR "Sierra Leone"[Title/Abstract] OR "Sierra Leonean"[Title/Abstract] OR "Somalia"[Title/Abstract] OR "Somali"[Title/Abstract] OR "South Africa"[Title/Abstract] OR "South African"[Title/Abstract] OR "South Sudan"[Title/Abstract] OR "South Sudanese"[Title/Abstract] OR "Sudan"[Title/Abstract] OR "Sudanese"[Title/Abstract] OR "Tanzania"[Title/Abstract] OR "Tanzanians"[Title/Abstract] OR "Togo"[Title/Abstract] OR "Togolese"[Title/Abstract] OR "Uganda"[Title/Abstract] OR "Ugandans"[Title/Abstract] OR "Zambia"[Title/Abstract] OR "Zambians"[Title/Abstract] OR "Zimbabwe"[Title/Abstract] OR "Zimbabweans"[Title/Abstract] OR "ivory coast"[Title/Abstract] OR "sub-Saharan african"[Title/Abstract] OR "subsaharan african"[Title/Abstract] OR "East African"[Title/Abstract] OR "Eastern African"[Title/Abstract] OR "Central African"[Title/Abstract] OR "north african"[Title/Abstract] OR "northern african"[Title/Abstract] OR "southern african"[Title/Abstract] OR "west african"[Title/Abstract] OR "western african"[Title/Abstract] OR "africa south of the sahara"[Title/Abstract] OR "sub-Saharan africa"[Title/Abstract] OR "subsaharan africa"[Title/Abstract] OR "central africa"[Title/Abstract] OR "north africa"[Title/Abstract] OR "northern africa"[Title/Abstract] OR "sahara"[Title/Abstract] OR "southern africa"[Title/Abstract] OR "east africa"[Title/Abstract] OR "eastern africa"[Title/Abstract] OR "west africa"[Title/Abstract] OR "western africa"[Title/Abstract]) | 518,510 |
| **Final** Combined | #1 AND #2 AND #3 AND  Limit to (English language AND yr="2015 -Current") | | (("Anemia during pregnancy"[Title/Abstract] OR "Maternal anemia"[Title/Abstract] OR "Anemia"[Title/Abstract] OR "Maternal anaemia"[Title/Abstract] OR "anaemia"[Title/Abstract] OR "Hemoglobin"[Title/Abstract] OR "maternal hemoglobin"[Title/Abstract] OR "Haemoglobin"[Title/Abstract] OR "Maternal Haemoglobin"[Title/Abstract] OR "Hematocrit"[Title/Abstract] OR "Maternal hematocrit"[Title/Abstract] OR "Haematocrit"[Title/Abstract] OR "Maternal haematocrit"[Title/Abstract] OR "Maternal hemoglobin level"[Title/Abstract] OR "Maternal hemoglobin concentration"[Title/Abstract] OR "Anemia"[MeSH Terms] OR "anemia, iron deficiency"[MeSH Terms] OR "Hemoglobins"[MeSH Terms] OR "Hematocrit"[MeSH Terms]) AND ("Low birth weight"[Title/Abstract] OR "Adverse birth outcome"[Title/Abstract] OR "Perinatal outcome"[Title/Abstract] OR "Birth outcome"[Title/Abstract] OR "birth weight"[Title/Abstract] OR "low for gestational age"[Title/Abstract] OR "infant, low birth weight"[MeSH Terms] OR "infant, extremely low birth weight"[MeSH Terms] OR "infant, very low birth weight"[MeSH Terms] OR "birth weight"[MeSH Terms]) AND ("Angola"[MeSH Terms] OR "Benin"[MeSH Terms] OR "Botswana"[MeSH Terms] OR "Burkina Faso"[MeSH Terms] OR "Burundi"[MeSH Terms] OR "Cabo Verde"[MeSH Terms] OR "Cameroon"[MeSH Terms] OR "Central African Republic"[MeSH Terms] OR "Chad"[MeSH Terms] OR "Comoros"[MeSH Terms] OR "Democratic Republic of the Congo"[MeSH Terms] OR "cote d ivoire"[MeSH Terms] OR "Equatorial Guinea"[MeSH Terms] OR "Eritrea"[MeSH Terms] OR "Eswatini"[MeSH Terms] OR "Ethiopia"[MeSH Terms] OR "Gabon"[MeSH Terms] OR "Gambia"[MeSH Terms] OR "Ghana"[MeSH Terms] OR "Guinea"[MeSH Terms] OR "Guinea-Bissau"[MeSH Terms] OR "Kenya"[MeSH Terms] OR "Lesotho"[MeSH Terms] OR "Liberia"[MeSH Terms] OR "Madagascar"[MeSH Terms] OR "Malawi"[MeSH Terms] OR "Mali"[MeSH Terms] OR "Mauritania"[MeSH Terms] OR "Mauritius"[MeSH Terms] OR "Mozambique"[MeSH Terms] OR "Namibia"[MeSH Terms] OR "Niger"[MeSH Terms] OR "Nigeria"[MeSH Terms] OR "Rwanda"[MeSH Terms] OR "Sao Tome and Principe"[MeSH Terms] OR "Senegal"[MeSH Terms] OR "Seychelles"[MeSH Terms] OR "Sierra Leone"[MeSH Terms] OR "Somalia"[MeSH Terms] OR "South Africa"[MeSH Terms] OR "South Sudan"[MeSH Terms] OR "Sudan"[MeSH Terms] OR "Tanzania"[MeSH Terms] OR "Togo"[MeSH Terms] OR "Uganda"[MeSH Terms] OR "Zambia"[MeSH Terms] OR "Zimbabwe"[MeSH Terms] OR "africa south of the sahara"[MeSH Terms:noexp] OR "africa, central"[MeSH Terms:noexp] OR "africa, northern"[MeSH Terms:noexp] OR "africa, southern"[MeSH Terms:noexp] OR "africa, eastern"[MeSH Terms:noexp] OR "africa, western"[MeSH Terms:noexp] OR ((("Angola"[Title/Abstract] OR "Angolan"[Title/Abstract] OR "Benin"[Title/Abstract] OR "Beninese"[Title/Abstract] OR "Botswana"[Title/Abstract] OR "Botswanan"[Title/Abstract] OR "Burkina Faso"[Title/Abstract] OR "Burkinabe"[Title/Abstract] OR "Burundi"[Title/Abstract] OR "Burundian"[Title/Abstract] OR "Cape Verde"[Title/Abstract] OR "Cape Verdean"[Title/Abstract] OR "Cameroon"[Title/Abstract] OR "Cameroonian"[Title/Abstract] OR "Central African Republic"[Title/Abstract] OR "Central African"[Title/Abstract] OR "Chad"[Title/Abstract] OR "Chadian"[Title/Abstract] OR "Comoros"[Title/Abstract] OR "Comorian"[Title/Abstract] OR "Democratic Republic of the Congo"[Title/Abstract] OR "Congolese"[Title/Abstract] OR "cote d ivoire"[Title/Abstract] OR "Ivorian"[Title/Abstract] OR "Equatorial Guinea"[Title/Abstract] OR "Equatorial Guinean"[Title/Abstract] OR "Eritrea"[Title/Abstract] OR "Eritrean"[Title/Abstract] OR "Eswatini"[Title/Abstract] OR "Swazi"[Title/Abstract] OR "Ethiopia"[Title/Abstract] OR "Ethiopian"[Title/Abstract] OR "Gabon"[Title/Abstract] OR "Gabonese"[Title/Abstract] OR "Gambia"[Title/Abstract] OR "Gambian"[Title/Abstract] OR "Ghana"[Title/Abstract] OR "Ghanaian"[Title/Abstract] OR "Guinea"[Title/Abstract] OR "Guinean"[Title/Abstract] OR "Guinea-Bissau"[Title/Abstract) OR "Guinea-Bissauan"[Title/Abstract]) OR "Kenya"[Title/Abstract] OR "Kenyan"[Title/Abstract] OR "Lesotho"[Title/Abstract] OR "Mosotho"[Title/Abstract] OR "Liberia"[Title/Abstract] OR "Liberian"[Title/Abstract] OR "Madagascar"[Title/Abstract] OR "Malagasy"[Title/Abstract] OR "Malawi"[Title/Abstract] OR "Malawian"[Title/Abstract] OR "Mali"[Title/Abstract] OR "Malian"[Title/Abstract] OR "Mauritania"[Title/Abstract] OR "Mauritanian"[Title/Abstract] OR "Mauritius"[Title/Abstract] OR "Mauritian"[Title/Abstract] OR "Mozambique"[Title/Abstract] OR "Mozambican"[Title/Abstract] OR "Namibia"[Title/Abstract] OR "Namibian"[Title/Abstract] OR "Niger"[Title/Abstract] OR "Nigerien"[Title/Abstract] OR "Nigeria"[Title/Abstract] OR "Nigerian"[Title/Abstract] OR "Rwanda"[Title/Abstract] OR "Rwandan"[Title/Abstract] OR "Sao Tome and Principe"[Title/Abstract] OR "Sao Tomeans"[Title/Abstract] OR "Santomeans"[Title/Abstract] OR "Senegal"[Title/Abstract] OR "Senegalese"[Title/Abstract] OR "Seychelles"[Title/Abstract] OR "Seychellois"[Title/Abstract] OR "Sierra Leone"[Title/Abstract] OR "Sierra Leonean"[Title/Abstract] OR "Somalia"[Title/Abstract] OR "Somali"[Title/Abstract] OR "South Africa"[Title/Abstract] OR "South African"[Title/Abstract] OR "South Sudan"[Title/Abstract] OR "South Sudanese"[Title/Abstract] OR "Sudan"[Title/Abstract] OR "Sudanese"[Title/Abstract] OR "Tanzania"[Title/Abstract] OR "Tanzanians"[Title/Abstract] OR "Togo"[Title/Abstract] OR "Togolese"[Title/Abstract] OR "Uganda"[Title/Abstract] OR "Ugandans"[Title/Abstract] OR "Zambia"[Title/Abstract] OR "Zambians"[Title/Abstract] OR "Zimbabwe"[Title/Abstract] OR "Zimbabweans"[Title/Abstract] OR "ivory coast"[Title/Abstract] OR "sub-Saharan african"[Title/Abstract] OR "subsaharan african"[Title/Abstract] OR "East African"[Title/Abstract] OR "Eastern African"[Title/Abstract] OR "Central African"[Title/Abstract] OR "north african"[Title/Abstract] OR "northern african"[Title/Abstract] OR "southern african"[Title/Abstract] OR "west african"[Title/Abstract] OR "western african"[Title/Abstract] OR "africa south of the sahara"[Title/Abstract] OR "sub-Saharan africa"[Title/Abstract] OR "subsaharan africa"[Title/Abstract] OR "central africa"[Title/Abstract] OR "north africa"[Title/Abstract] OR "northern africa"[Title/Abstract] OR "sahara"[Title/Abstract] OR "southern africa"[Title/Abstract] OR "east africa"[Title/Abstract] OR "eastern africa"[Title/Abstract] OR "west africa"[Title/Abstract] OR "western africa"[Title/Abstract]))) AND ((english[Filter]) AND (2015:2024[pdat])) | 311 |

| **Embase data base Final Searching Date: June 12, 2024** | | | | |
| --- | --- | --- | --- | --- |
| **Concept** | **Line** | **Search term** | **Search strategy** | **Records** |
| Anemia | #1 | Anemia during pregnancy, Maternal anemia, anemia, Maternal anaemia, anaemia, Hemoglobin, maternal hemoglobin, Haemoglobin, Maternal Haemoglobin, hematocrit, Maternal hematocrit, Haematocrit,  Maternal haematocrit, Maternal hemoglobin level,  Maternal hemoglobin concentration | exp Anemia/ OR exp anemia, iron deficiency/ OR exp Hemoglobins/ OR exp Hematocrit/ OR ("Anemia during pregnancy" OR "Maternal anemia" OR "Anemia" OR "Maternal anaemia" OR "anaemia" OR "Hemoglobin" OR "maternal hemoglobin" OR "Haemoglobin" OR "Maternal Haemoglobin" OR "Hematocrit" OR "Maternal hematocrit" OR "Haematocrit" OR "Maternal haematocrit" OR "Maternal hemoglobin level" OR “Maternal hemoglobin concentration”).ab,kw,ti. | 993,370 |
| Low birth weight | #2 | Low birth weight,  Adverse birth outcome, Perinatal outcome,  Birth outcome,  Birth weight,  Low for gestational age | exp infant, low birth weight/ OR exp infant, extremely low birth weight/ OR exp infant, very low birth weight/ OR exp birth weight/ OR (“Low birth weight" OR "Adverse birth outcome" OR "Perinatal outcome" OR "Birth outcome" OR "birth weight" OR "low for gestational age").ab,kw,ti. | 178,985 |
| Sub-Saharan African Countries | #3 | Angola, Benin, Botswana,  Burkina Faso, Burundi, Cape Verde, Cameroon, Central African Republic, Chad, Comoros, Democratic Republic of the Congo, Cote D'ivoire, Equatorial Guinea,  Eritrea, Eswatini, Ethiopia, Gabon, Gambia, Ghana, Guinea, Guinea-Bissau, Kenya, Lesotho, Liberia, Madagascar, Malawi, Mali, Mauritania, Mauritius, Mozambique, Namibia, Niger, Nigeria, Rwanda, Sao Tome and Principe, Senegal, Seychelles, Sierra Leone, Somalia, South Africa, South Sudan, Sudan, Tanzania, Togo, Uganda, Zambia, Zimbabwe, africa south of the sahara, Sub-Saharan africa, subsaharan africa, central africa, north africa, northern africa, sahara, southern africa, east africa, eastern africa, West africa, western africa | ((((exp 'Angola'/ or 'angola*':ti,ab,kw.mp. or exp 'Benin'/ or 'benin*':ti,ab,kw.mp. or exp 'Botswana'/ or 'botswana*':ti,ab,kw.mp. or exp 'Burkina Faso'/ or 'burkina fasso*':ti,ab,kw.mp. or 'burkinabe*':ti,ab,kw.mp. or 'burkinese*':ti,ab,kw.mp. or exp 'Burundi'/ or 'burundi*':ti,ab,kw.mp. or 'urundi*':ti,ab,kw.mp. or 'cabo verde*':ti,ab,kw.mp. or exp 'Cape Verde'/ or 'cape verde*':ti,ab,kw.mp. or exp 'Cameroon'/ or 'cameroon*':ti,ab,kw.mp. or 'cameron':ti,ab,kw.mp. or 'cameroun':ti,ab,kw.mp. or exp 'Central African Republic'/ or 'central african republic':ti,ab,kw.mp. or 'central african*':ti,ab,kw.mp. or exp 'Chad'/ or 'chad*':ti,ab,kw.mp. or exp 'Comoros'/ or 'comoros':ti,ab,kw.mp. or 'comoro islands':ti,ab,kw.mp. or 'iles comores':ti,ab,kw.mp. or 'comorian*':ti,ab,kw.mp. or 'democratic republic of the congo':ti,ab,kw.mp. or exp 'Democratic Republic Congo'/ or exp 'congo'/ or 'congo*':ti,ab,kw.mp. or exp 'cote d`Ivoire'/ or 'cote d`Ivoire':ti,ab,kw.mp. or 'ivory coast':ti,ab,kw.mp. or 'ivorian*':ti,ab,kw.mp. or exp 'Equatorial Guinea'/ or 'Equatorial Guinea*':ti,ab,kw.mp. or 'equatoguinean*':ti,ab,kw.mp. or exp 'Eritrea'/ or 'eritrea*':ti,ab,kw.mp. or exp 'Eswatini'/ or 'eswatini':ti,ab,kw.mp. or exp 'Ethiopia'/ or 'ethiopia*':ti,ab,kw.mp. or exp 'Gabon'/ or 'gabon*':ti,ab,kw.mp. or exp 'Gambia'/ or 'gambia*':ti,ab,kw.mp. or exp 'Ghana'/ or 'ghana*':ti,ab,kw.mp. or exp 'Guinea'/ or 'guinea*':ti,ab,kw.mp. or exp 'Guinea-Bissau'/ or 'Guinea-Bissau':ti,ab,kw.mp. or 'guinea bissau':ti,ab,kw.mp. or exp 'Kenya'/ or 'kenya*':ti,ab,kw.mp. or exp 'Lesotho'/ or 'lesotho*':ti,ab,kw.mp. or 'lesothan*':ti,ab,kw.mp. or exp 'Liberia'/ or 'liberia*':ti,ab,kw.mp. or exp 'Madagascar'/ or 'madagascar':ti,ab,kw.mp. or 'madagascan*':ti,ab,kw.mp. or exp 'Malawi'/ or 'malawi*':ti,ab,kw.mp. or exp 'Mali'/ or 'mali*':ti,ab,kw.mp. or exp 'Mauritania'/ or 'mauritania*':ti,ab,kw.mp. or exp 'Mauritius'/ or 'mauritius*':ti,ab,kw.mp. or 'mauritian*':ti,ab,kw.mp. or exp 'Mozambique'/ or 'mozambique':ti,ab,kw.mp. or 'mozambican*':ti,ab,kw.mp. or exp 'Namibia'/ or 'namibia*':ti,ab,kw.mp. or exp 'Niger'/ or 'niger*':ti,ab,kw.mp. or exp 'Nigeria'/ or 'nigeria*':ti,ab,kw.mp. or exp 'Rwanda'/ or 'rwanda*':ti,ab,kw.mp. or 'rwandese':ti,ab,kw.mp. or 'ruanda*':ti,ab,kw.mp. or 'ruandese':ti,ab,kw.mp. or exp 'Sao Tome/) and Principe'/) or 'sao tome.mp.) and principe':ti,ab,kw.mp.) or 'sao tomean*':ti,ab,kw.mp. or exp 'Senegal'/ or 'senegal*':ti,ab,kw.mp. or exp 'Seychelles'/ or 'seychell*':ti,ab,kw.mp. or exp 'Sierra Leone'/ or 'sierra leone*':ti,ab,kw.mp. or exp 'Somalia'/ or 'somali*':ti,ab,kw.mp. or exp 'South Africa'/ or 'south africa*':ti,ab,kw.mp. or exp 'South Sudan'/ or 'south sudan*':ti,ab,kw.mp. or exp 'Sudan'/ or 'sudan*':ti,ab,kw.mp. or exp 'Tanzania'/ or 'tanzania*':ti,ab,kw.mp. or 'tanganyika*':ti,ab,kw.mp. or exp 'Togo'/ or 'togo*':ti,ab,kw.mp. or 'togolese republic':ti,ab,kw.mp. or exp 'Uganda'/ or 'uganda*':ti,ab,kw.mp. or exp 'Zambia'/ or 'zambia*':ti,ab,kw.mp. or exp 'Zimbabwe'/ or 'zimbabwe*':ti,ab,kw.mp. or 'northern rhodesia*':ti,ab,kw.mp. or exp 'Africa south of the Sahara'/ or 'africa south of the sahara':ti,ab,kw.mp. or 'sub sahara africa':ti,ab,kw.mp. or 'subsaharan africa':ti,ab,kw.mp. or exp 'Central Africa'/ or 'central africa':ti,ab,kw.mp. or 'africa, central':ti,ab,kw.mp. or 'africa, northern':ti,ab,kw.mp. or 'north africa':ti,ab,kw.mp. or 'northern africa':ti,ab,kw.mp. or 'magreb':ti,ab,kw.mp. or 'maghrib':ti,ab,kw.mp. or 'sahara':ti,ab,kw.mp. or 'africa, southern':ti,ab,kw.mp. or 'africa, southern':ti,ab,kw.mp. or 'southern africa*':ti,ab,kw.mp. or 'africa, eastern':ti,ab,kw.mp. or 'east africa*':ti,ab,kw.mp. or 'eastern africa*':ti,ab,kw.mp. or 'africa, western':ti,ab,kw.mp. or 'west africa*':ti,ab,kw.mp. or 'western africa*':ti,ab,kw.mp. | 325,129 |
| **Final** combined | #1 AND #2 AND #3 | | (exp Anemia/ OR exp anemia, iron deficiency/ OR exp Hemoglobins/ OR exp Hematocrit/ OR ("Anemia during pregnancy" OR "Maternal anemia" OR "Anemia" OR "Maternal anaemia" OR "anaemia" OR "Hemoglobin" OR "maternal hemoglobin" OR "Haemoglobin" OR "Maternal Haemoglobin" OR "Hematocrit" OR "Maternal hematocrit" OR "Haematocrit" OR "Maternal haematocrit" OR "Maternal hemoglobin level" OR “Maternal hemoglobin concentration”).ab,kw,ti.) and (exp infant, low birth weight/ OR exp infant, extremely low birth weight/ OR exp infant, very low birth weight/ OR exp birth weight/ OR (“Low birth weight" OR "Adverse birth outcome" OR "Perinatal outcome" OR "Birth outcome" OR "birth weight" OR "low for gestational age").ab,kw,ti.) and (((((exp 'Angola'/ or 'angola*':ti,ab,kw.mp. or exp 'Benin'/ or 'benin*':ti,ab,kw.mp. or exp 'Botswana'/ or 'botswana*':ti,ab,kw.mp. or exp 'Burkina Faso'/ or 'burkina fasso*':ti,ab,kw.mp. or 'burkinabe*':ti,ab,kw.mp. or 'burkinese*':ti,ab,kw.mp. or exp 'Burundi'/ or 'burundi*':ti,ab,kw.mp. or 'urundi*':ti,ab,kw.mp. or 'cabo verde*':ti,ab,kw.mp. or exp 'Cape Verde'/ or 'cape verde*':ti,ab,kw.mp. or exp 'Cameroon'/ or 'cameroon*':ti,ab,kw.mp. or 'cameron':ti,ab,kw.mp. or 'cameroun':ti,ab,kw.mp. or exp 'Central African Republic'/ or 'central african republic':ti,ab,kw.mp. or 'central african*':ti,ab,kw.mp. or exp 'Chad'/ or 'chad*':ti,ab,kw.mp. or exp 'Comoros'/ or 'comoros':ti,ab,kw.mp. or 'comoro islands':ti,ab,kw.mp. or 'iles comores':ti,ab,kw.mp. or 'comorian*':ti,ab,kw.mp. or 'democratic republic of the congo':ti,ab,kw.mp. or exp 'Democratic Republic Congo'/ or exp 'congo'/ or 'congo*':ti,ab,kw.mp. or exp 'cote d`Ivoire'/ or 'cote d`Ivoire':ti,ab,kw.mp. or 'ivory coast':ti,ab,kw.mp. or 'ivorian*':ti,ab,kw.mp. or exp 'Equatorial Guinea'/ or 'Equatorial Guinea*':ti,ab,kw.mp. or 'equatoguinean*':ti,ab,kw.mp. or exp 'Eritrea'/ or 'eritrea*':ti,ab,kw.mp. or exp 'Eswatini'/ or 'eswatini':ti,ab,kw.mp. or exp 'Ethiopia'/ or 'ethiopia*':ti,ab,kw.mp. or exp 'Gabon'/ or 'gabon*':ti,ab,kw.mp. or exp 'Gambia'/ or 'gambia*':ti,ab,kw.mp. or exp 'Ghana'/ or 'ghana*':ti,ab,kw.mp. or exp 'Guinea'/ or 'guinea*':ti,ab,kw.mp. or exp 'Guinea-Bissau'/ or 'Guinea-Bissau':ti,ab,kw.mp. or 'guinea bissau':ti,ab,kw.mp. or exp 'Kenya'/ or 'kenya*':ti,ab,kw.mp. or exp 'Lesotho'/ or 'lesotho*':ti,ab,kw.mp. or 'lesothan*':ti,ab,kw.mp. or exp 'Liberia'/ or 'liberia*':ti,ab,kw.mp. or exp 'Madagascar'/ or 'madagascar':ti,ab,kw.mp. or 'madagascan*':ti,ab,kw.mp. or exp 'Malawi'/ or 'malawi*':ti,ab,kw.mp. or exp 'Mali'/ or 'mali*':ti,ab,kw.mp. or exp 'Mauritania'/ or 'mauritania*':ti,ab,kw.mp. or exp 'Mauritius'/ or 'mauritius*':ti,ab,kw.mp. or 'mauritian*':ti,ab,kw.mp. or exp 'Mozambique'/ or 'mozambique':ti,ab,kw.mp. or 'mozambican*':ti,ab,kw.mp. or exp 'Namibia'/ or 'namibia*':ti,ab,kw.mp. or exp 'Niger'/ or 'niger*':ti,ab,kw.mp. or exp 'Nigeria'/ or 'nigeria*':ti,ab,kw.mp. or exp 'Rwanda'/ or 'rwanda*':ti,ab,kw.mp. or 'rwandese':ti,ab,kw.mp. or 'ruanda*':ti,ab,kw.mp. or 'ruandese':ti,ab,kw.mp. or exp 'Sao Tome/) and Principe'/) or 'sao tome.mp.) and principe':ti,ab,kw.mp.) or 'sao tomean*':ti,ab,kw.mp. or exp 'Senegal'/ or 'senegal*':ti,ab,kw.mp. or exp 'Seychelles'/ or 'seychell*':ti,ab,kw.mp. or exp 'Sierra Leone'/ or 'sierra leone*':ti,ab,kw.mp. or exp 'Somalia'/ or 'somali*':ti,ab,kw.mp. or exp 'South Africa'/ or 'south africa*':ti,ab,kw.mp. or exp 'South Sudan'/ or 'south sudan*':ti,ab,kw.mp. or exp 'Sudan'/ or 'sudan*':ti,ab,kw.mp. or exp 'Tanzania'/ or 'tanzania*':ti,ab,kw.mp. or 'tanganyika*':ti,ab,kw.mp. or exp 'Togo'/ or 'togo*':ti,ab,kw.mp. or 'togolese republic':ti,ab,kw.mp. or exp 'Uganda'/ or 'uganda*':ti,ab,kw.mp. or exp 'Zambia'/ or 'zambia*':ti,ab,kw.mp. or exp 'Zimbabwe'/ or 'zimbabwe*':ti,ab,kw.mp. or 'northern rhodesia*':ti,ab,kw.mp. or exp 'Africa south of the Sahara'/ or 'africa south of the sahara':ti,ab,kw.mp. or 'sub sahara africa':ti,ab,kw.mp. or 'subsaharan africa':ti,ab,kw.mp. or exp 'Central Africa'/ or 'central africa':ti,ab,kw.mp. or 'africa, central':ti,ab,kw.mp. or 'africa, northern':ti,ab,kw.mp. or 'north africa':ti,ab,kw.mp. or 'northern africa':ti,ab,kw.mp. or 'magreb':ti,ab,kw.mp. or 'maghrib':ti,ab,kw.mp. or 'sahara':ti,ab,kw.mp. or 'africa, southern':ti,ab,kw.mp. or 'africa, southern':ti,ab,kw.mp. or 'southern africa*':ti,ab,kw.mp. or 'africa, eastern':ti,ab,kw.mp. or 'east africa*':ti,ab,kw.mp. or 'eastern africa*':ti,ab,kw.mp. or 'africa, western':ti,ab,kw.mp. or 'west africa*':ti,ab,kw.mp. or 'western africa*':ti,ab,kw.mp.) | 849 |
| Filters | limit to (English language and yr="2015 -Current" and article) | | | 367 |

| **Cochrane Library** **Final Searching Date: June 12, 2024** | | | | | |
| --- | --- | --- | --- | --- | --- |
| **Concept** | **Line** | **Search term** | | **Search strategy** | **Records** |
| Anemia | #1 | Anemia during pregnancy, Maternal anemia, anemia, Maternal anaemia, anaemia, Hemoglobin, maternal hemoglobin, Haemoglobin, Maternal Haemoglobin, hematocrit, Maternal hematocrit, Haematocrit,  Maternal haematocrit, Maternal hemoglobin level,  Maternal hemoglobin concentration | | [mh "Anemia"] OR [mh "anemia, iron deficiency"] OR [mh "Hemoglobins"] OR [mh "Hematocrit"] OR ("Anemia during pregnancy" OR "Maternal anemia" OR "Anemia" OR "Maternal anaemia" OR "anaemia" OR "Hemoglobin" OR "maternal hemoglobin" OR "Haemoglobin" OR "Maternal Haemoglobin" OR "Hematocrit" OR "Maternal hematocrit" OR "Haematocrit" OR "Maternal haematocrit" OR "Maternal hemoglobin level" OR “Maternal hemoglobin concentration”):ti,ab,kw | 68,211 |
| Low birth weight | #2 | Low birth weight,  Adverse birth outcome, Perinatal outcome,  Birth outcome,  Birth weight,  Low for gestational age | | [mh "infant, low birth weight"] OR [mh "infant, extremely low birth weight"] OR [mh "infant, very low birth weight"] OR [mh "birth weight"] OR ("Low birth weight" OR "Adverse birth outcome" OR "Perinatal outcome" OR "Birth outcome" OR "birth weight" OR "low for gestational age"):ti,ab,kw | 14,110 |
| Sub-Saharan African Countries | #3 | Angola, Benin, Botswana,  Burkina Faso, Burundi, Cape Verde, Cameroon, Central African Republic, Chad, Comoros, Democratic Republic of the Congo, Cote D'ivoire, Equatorial Guinea,  Eritrea, Eswatini, Ethiopia, Gabon, Gambia, Ghana, Guinea, Guinea-Bissau, Kenya, Lesotho, Liberia, Madagascar, Malawi, Mali, Mauritania, Mauritius, Mozambique, Namibia, Niger, Nigeria, Rwanda, Sao Tome and Principe, Senegal, Seychelles, Sierra Leone, Somalia, South Africa, South Sudan, Sudan, Tanzania, Togo, Uganda, Zambia, Zimbabwe, africa south of the sahara, Sub-Saharan africa, subsaharan africa, central africa, north africa, northern africa, sahara, southern africa, east africa, eastern africa, West africa, western africa | | [mh ^“angola”] OR [mh ^“benin”] OR [mh ^“Botswana”] OR [mh ^“burkina faso”] OR [mh ^“burundi”] OR [mh ^“cabo verde”] OR [mh ^“Cameroon”] OR [mh ^“central african republic”] OR [mh ^“chad”] OR [mh ^“comoros”] OR [mh ^“democratic republic of the congo”] OR [mh ^“congo”] OR [mh ^“cote d’ivoire”] OR [mh ^“equatorial guinea”] OR [mh ^“Eritrea”] OR [mh ^“Eswatini”] OR [mh ^“Ethiopia”] OR [mh ^“gabon”] OR [mh ^“gambia”] OR [mh ^“ghana”] OR [mh ^“Guinea”] OR [mh ^“Guinea-Bissau”] OR [mh ^"Kenya"] OR [mh ^“Lesotho”] OR [mh ^“Liberia”] OR [mh ^“Madagascar”] OR [mh ^“Malawi”] OR [mh ^“Mali”] OR [mh ^“Mauritania”] OR [mh ^“Mauritius”] OR [mh ^“Mozambique”] OR [mh ^“Namibia”] OR [mh ^“Niger”] OR [mh ^“Nigeria”] OR [mh ^“Rwanda”] OR [mh ^“Sao Tome and Principe”] OR [mh ^“Senegal”] OR [mh ^“Seychelles”] OR [mh ^“Sierra Leone”] OR [mh ^“Somalia”] OR [mh ^“South Africa”] OR [mh ^“South Sudan”] OR [mh ^“Sudan”] OR [mh ^“Tanzania”] OR [mh ^“Togo”] OR [mh ^“Uganda”] OR [mh ^“Zambia”] OR [mh ^“Zimbabwe”] OR [mh ^“Africa south of the Sahara”] OR [mh ^“Central Africa”] OR (angola OR benin OR "burkina faso" OR "burkina fasso" OR burundi OR urundi OR "cabo verde" OR "cape verde" OR cameroon OR cameron OR cameroun OR "central african republic" OR chad OR comoros OR "democratic republic of the congo" OR "democratic republic congo" OR congo OR zaire OR "cote d’ivoire" OR "cote d’ ivoire" OR "cote divoire" OR "cote d ivoire" OR "ivory coast" OR "equatorial guinea" OR eritrea OR eswatini OR ethiopia OR gabon OR "gabonese republic" OR gambia OR ghana OR guinea OR "guinea bissau" OR Kenya OR lesotho OR Liberia OR madagascar OR malawi OR mali OR mauritania OR mauritius OR mozambique OR namibia OR niger OR nigeria OR rwanda OR ruanda OR "sao tome and principe" OR senegal OR seychelles OR "sierra leone" OR somalia OR "south africa" OR "south sudan" OR sudan OR tanzania OR tanganyika OR togo OR "togolese republic" OR uganda OR zambia OR zimbabwe OR "africa south of the sahara" OR "sub saharan africa" OR "subsaharan africa" OR "africa, central" OR "central africa" OR "africa, northern" OR "north africa" OR "northern africa" OR sahara OR "africa, southern" OR "southern africa" OR "africa, eastern" OR "east africa" OR "eastern africa" OR "africa, western" OR "west africa" OR "western africa"):ti,ab,kw OR (Angolan OR beninese OR benineses OR botswana OR batswana OR burkinabe OR burkinese OR burundian OR burundians OR "cape verdean" OR "cape verdeans" OR "cabo verdean" OR "cabo verdeans" OR cameroonian OR cameroonians OR "central african" OR "central africans" OR chadian OR chadians OR comorian OR comorians OR congolese OR ivorian OR ivorians OR "equatorial guinean" OR "equatorial guineans" OR equatoguinean OR equatoguineans OR eritrean OR eritreans OR ethiopian OR ethiopians OR gabonese OR gabonaise OR gambian OR gambians OR ghanaian OR ghanaians OR guinean OR guineans OR "bissau guinean" OR "bissau guineans" OR kenyan OR kenyans OR lesothan OR lesothans OR lesothonian OR lesothonians OR liberian OR liberians OR madagascan OR madagascans OR malawian OR malawians OR malian OR malians OR mauritanian OR mauritanians OR mauritian OR mauritians OR mozambican OR mozambicans OR namibian OR namibians OR nigerien OR nigeriens OR nigerian OR nigerians OR rwandan OR rwandans OR rwandese OR ruandan OR ruandans OR ruandese OR "sao tomean" OR "sao tomeans" OR santomean OR santomeans OR senegalese OR seychellois OR seychelloise OR seychelloises OR "sierra leonean" OR "sierra leoneans" OR somali OR somalis OR "south african" OR "south africans" OR "south sudanese" OR sudanese OR tanzanian OR tanzanians OR tanganyikan OR tanganyikans OR ugandan OR ugandans OR zambian OR zambians OR zimbabwean OR Zimbabweans OR "sub-Saharan african" OR "subsaharan african" OR "East African" OR "Eastern African" OR "Central African" OR "southern african" OR "west african" OR "western african" OR "africa south of the sahara" OR "sub-Saharan africa" OR "subsaharan africa" OR "central african" OR "saharan" OR "southern african" OR "east african" OR "eastern african" OR "west african" OR "western african"):ti,ab,kw | 25,733 |
| **Final** Combined | #1 AND #2 AND #3 | | ([mh "Anemia"] OR [mh "anemia, iron deficiency"] OR [mh "Hemoglobins"] OR [mh "Hematocrit"] OR ("Anemia during pregnancy" OR "Maternal anemia" OR "Anemia" OR "Maternal anaemia" OR "anaemia" OR "Hemoglobin" OR "maternal hemoglobin" OR "Haemoglobin" OR "Maternal Haemoglobin" OR "Hematocrit" OR "Maternal hematocrit" OR "Haematocrit" OR "Maternal haematocrit" OR "Maternal hemoglobin level" OR “Maternal hemoglobin concentration”):ti,ab,kw) AND ([mh "infant, low birth weight"] OR [mh "infant, extremely low birth weight"] OR [mh "infant, very low birth weight"] OR [mh "birth weight"] OR ("Low birth weight" OR "Adverse birth outcome" OR "Perinatal outcome" OR "Birth outcome" OR "birth weight" OR "low for gestational age"):ti,ab,kw) AND ([mh ^“angola”] OR [mh ^“benin”] OR [mh ^“Botswana”] OR [mh ^“burkina faso”] OR [mh ^“burundi”] OR [mh ^“cabo verde”] OR [mh ^“Cameroon”] OR [mh ^“central african republic”] OR [mh ^“chad”] OR [mh ^“comoros”] OR [mh ^“democratic republic of the congo”] OR [mh ^“congo”] OR [mh ^“cote d’ivoire”] OR [mh ^“equatorial guinea”] OR [mh ^“Eritrea”] OR [mh ^“Eswatini”] OR [mh ^“Ethiopia”] OR [mh ^“gabon”] OR [mh ^“gambia”] OR [mh ^“ghana”] OR [mh ^“Guinea”] OR [mh ^“Guinea-Bissau”] OR [mh ^"Kenya"] OR [mh ^“Lesotho”] OR [mh ^“Liberia”] OR [mh ^“Madagascar”] OR [mh ^“Malawi”] OR [mh ^“Mali”] OR [mh ^“Mauritania”] OR [mh ^“Mauritius”] OR [mh ^“Mozambique”] OR [mh ^“Namibia”] OR [mh ^“Niger”] OR [mh ^“Nigeria”] OR [mh ^“Rwanda”] OR [mh ^“Sao Tome and Principe”] OR [mh ^“Senegal”] OR [mh ^“Seychelles”] OR [mh ^“Sierra Leone”] OR [mh ^“Somalia”] OR [mh ^“South Africa”] OR [mh ^“South Sudan”] OR [mh ^“Sudan”] OR [mh ^“Tanzania”] OR [mh ^“Togo”] OR [mh ^“Uganda”] OR [mh ^“Zambia”] OR [mh ^“Zimbabwe”] OR [mh ^“Africa south of the Sahara”] OR [mh ^“Central Africa”] OR (angola OR benin OR "burkina faso" OR "burkina fasso" OR burundi OR urundi OR "cabo verde" OR "cape verde" OR cameroon OR cameron OR cameroun OR "central african republic" OR chad OR comoros OR "democratic republic of the congo" OR "democratic republic congo" OR congo OR zaire OR "cote d’ivoire" OR "cote d’ ivoire" OR "cote divoire" OR "cote d ivoire" OR "ivory coast" OR "equatorial guinea" OR eritrea OR eswatini OR ethiopia OR gabon OR "gabonese republic" OR gambia OR ghana OR guinea OR "guinea bissau" OR Kenya OR lesotho OR Liberia OR madagascar OR malawi OR mali OR mauritania OR mauritius OR mozambique OR namibia OR niger OR nigeria OR rwanda OR ruanda OR "sao tome and principe" OR senegal OR seychelles OR "sierra leone" OR somalia OR "south africa" OR "south sudan" OR sudan OR tanzania OR tanganyika OR togo OR "togolese republic" OR uganda OR zambia OR zimbabwe OR "africa south of the sahara" OR "sub saharan africa" OR "subsaharan africa" OR "africa, central" OR "central africa" OR "africa, northern" OR "north africa" OR "northern africa" OR sahara OR "africa, southern" OR "southern africa" OR "africa, eastern" OR "east africa" OR "eastern africa" OR "africa, western" OR "west africa" OR "western africa"):ti,ab,kw OR ("Angolan" OR beninese OR benineses OR botswana OR batswana OR burkinabe OR burkinese OR burundian OR burundians OR "cape verdean" OR "cape verdeans" OR "cabo verdean" OR "cabo verdeans" OR cameroonian OR cameroonians OR "central african" OR "central africans" OR chadian OR chadians OR comorian OR comorians OR congolese OR ivorian OR ivorians OR "equatorial guinean" OR "equatorial guineans" OR equatoguinean OR equatoguineans OR eritrean OR eritreans OR ethiopian OR ethiopians OR gabonese OR gabonaise OR gambian OR gambians OR ghanaian OR ghanaians OR guinean OR guineans OR "bissau guinean" OR "bissau guineans" OR kenyan OR kenyans OR lesothan OR lesothans OR lesothonian OR lesothonians OR liberian OR liberians OR madagascan OR madagascans OR malawian OR malawians OR malian OR malians OR mauritanian OR mauritanians OR mauritian OR mauritians OR mozambican OR mozambicans OR namibian OR namibians OR nigerien OR nigeriens OR nigerian OR nigerians OR rwandan OR rwandans OR rwandese OR ruandan OR ruandans OR ruandese OR "sao tomean" OR "sao tomeans" OR santomean OR santomeans OR senegalese OR seychellois OR seychelloise OR seychelloises OR "sierra leonean" OR "sierra leoneans" OR somali OR somalis OR "south african" OR "south africans" OR "south sudanese" OR sudanese OR tanzanian OR tanzanians OR tanganyikan OR tanganyikans OR ugandan OR ugandans OR zambian OR zambians OR zimbabwean OR Zimbabweans OR "sub-Saharan african" OR "subsaharan african" OR "East African" OR "Eastern African" OR "Central African" OR "southern african" OR "west african" OR "western african" OR "africa south of the sahara" OR "sub-Saharan africa" OR "subsaharan africa" OR "central african" OR "saharan" OR "southern african" OR "east african" OR "eastern african" OR "west african" OR "western african"):ti,ab,kw) | | 236 |
| Filters | limit to (English language and yr="2015 -Current" and article) | | | | 170 |

| **Scopus Data base** **Final Searching Date: June 12, 2024** | | | | |
| --- | --- | --- | --- | --- |
| **Concept** | **Line** | **Search term** | **Search strategy** | **Records** |
| Anemia | #1 | Anemia during pregnancy, Maternal anemia, anemia, Maternal anaemia, anaemia, Hemoglobin, maternal hemoglobin, Haemoglobin, Maternal Haemoglobin, hematocrit, Maternal hematocrit, Haematocrit,  Maternal haematocrit, Maternal hemoglobin level,  Maternal hemoglobin concentration | INDEXTERMS ( "Anemia" OR "anemia, iron deficiency" OR "Hemoglobins" OR "Hematocrit" ) OR TITLE-ABS-KEY ( "Anemia during pregnancy" OR "Maternal anemia" OR "Anemia" OR "Maternal anaemia" OR "anaemia" OR "Hemoglobin" OR "maternal hemoglobin" OR "Haemoglobin" OR "Maternal Haemoglobin" OR "Hematocrit" OR "Maternal hematocrit" OR "Haematocrit" OR "Maternal haematocrit" OR "Maternal hemoglobin level" OR "Maternal hemoglobin concentration" ) | 845,539 |
| Low birth weight | #2 | Low birth weight,  Adverse birth outcome, Perinatal outcome,  Birth outcome,  Birth weight,  Low for gestational age | INDEXTERMS ("infant, low birth weight" OR "infant, extremely low birth weight" OR "infant, very low birth weight" OR "birth weight") OR TITLE-ABS-KEY ("Low birth weight" OR "Adverse birth outcome" OR "Perinatal outcome" OR "Birth outcome" OR "birth weight" OR "low for gestational age") | 174,740 |
| Sub-Saharan African Countries | #3 | Angola, Benin, Botswana,  Burkina Faso, Burundi, Cape Verde, Cameroon, Central African Republic, Chad, Comoros, Democratic Republic of the Congo, Cote D'ivoire, Equatorial Guinea,  Eritrea, Eswatini, Ethiopia, Gabon, Gambia, Ghana, Guinea, Guinea-Bissau, Kenya, Lesotho, Liberia, Madagascar, Malawi, Mali, Mauritania, Mauritius, Mozambique, Namibia, Niger, Nigeria, Rwanda, Sao Tome and Principe, Senegal, Seychelles, Sierra Leone, Somalia, South Africa, South Sudan, Sudan, Tanzania, Togo, Uganda, Zambia, Zimbabwe, africa south of the sahara, Sub-Saharan africa, subsaharan africa, central africa, north africa, northern africa, sahara, southern africa, east africa, eastern africa, West africa, western africa | INDEXTERMS ( "Angola" OR "Benin" OR "Botswana" OR "Burkina Faso" OR "Burundi" OR "Cape Verde" OR "Cameroon" OR "Central African Republic" OR "Chad" OR "Comoros" OR "Democratic Republic Congo" OR "congo" OR "cote d` Ivoire" OR "Equatorial Guinea" OR "Eritrea" OR "Eswatini" OR "Ethiopia" OR "Gabon" OR "Gambia" OR "Ghana" OR "Guinea" OR "Guinea-Bissau" OR "Kenya" OR "Lesotho" OR "Liberia" OR "Madagascar" OR "Malawi" OR "Mali" OR "Mauritania" OR "Mauritius" OR "Mozambique" OR "Namibia" OR "Niger" OR "Nigeria" OR "Rwanda" OR "Sao Tome and Principe" OR "Senegal" OR "Seychelles" OR "Somalia" OR "South Africa" OR "South Sudan" OR "Sudan" OR "Tanzania" OR "Togo" OR "Uganda" OR "Zambia" OR "Zimbabwe" OR "Africa south of the Sahara" OR "Central Africa" ) OR TITLE-ABS-KEY ( angola OR benin OR "burkina faso" OR "burkina fasso" OR burundi OR urundi OR "cabo verde" OR "cape verde" OR cameroon OR cameron OR cameroun OR "central african republic" OR chad OR comoros OR "democratic republic of the congo" OR "democratic republic congo" OR congo OR zaire OR "cote d'ivoire" OR "cote d' ivoire" OR "cote divoire" OR "cote d ivoire" OR "ivory coast" OR "equatorial guinea" OR eritrea OR eswatini OR ethiopia OR gabon OR "gabonese republic" OR gambia OR ghana OR guinea OR "guinea bissau" OR kenya OR lesotho OR liberia OR madagascar OR malawi OR mali OR mauritania OR mauritius OR mozambique OR namibia OR niger OR nigeria OR rwanda OR ruanda OR "sao tome and principe" OR senegal OR seychelles OR "sierra leone" OR somalia OR "south africa" OR "south sudan" OR sudan OR tanzania OR tanganyika OR togo OR "togolese republic" OR uganda OR zambia OR zimbabwe OR "africa south of the sahara" OR "sub saharan africa" OR "subsaharan africa" OR "africa, central" OR "central africa" OR "africa, northern" OR "north africa" OR "northern africa" OR sahara OR "africa, southern" OR "southern africa" OR "africa, eastern" OR "east africa" OR "eastern africa" OR "africa, western" OR "west africa" OR "western africa" ) OR TITLE-ABS-KEY ( angolan OR beninese OR benineses OR botswana OR batswana OR burkinabe OR burkinese OR burundian OR burundians OR "cape verdean" OR "cape verdeans" OR "cabo verdean" OR "cabo verdeans" OR cameroonian OR cameroonians OR "central african" OR "central africans" OR chadian OR chadians OR comorian OR comorians OR congolese OR ivorian OR ivorians OR "equatorial guinean" OR "equatorial guineans" OR equatoguinean OR equatoguineans OR eritrean OR eritreans OR ethiopian OR ethiopians OR gabonese OR gabonaise OR gambian OR gambians OR ghanaian OR ghanaians OR guinean OR guineans OR "bissau guinean" OR "bissau guineans" OR kenyan OR kenyans OR lesothan OR lesothans OR lesothonian OR lesothonians OR liberian OR liberians OR madagascan OR madagascans OR malawian OR malawians OR malian OR malians OR mauritanian OR mauritanians OR mauritian OR mauritians OR mozambican OR mozambicans OR namibian OR namibians OR nigerien OR nigeriens OR nigerian OR nigerians OR rwandan OR rwandans OR rwandese OR ruandan OR ruandans OR ruandese OR "sao tomean" OR "sao tomeans" OR santomean OR santomeans OR senegalese OR seychellois OR seychelloise OR seychelloises OR "sierra leonean" OR "sierra leoneans" OR somali OR somalis OR "south african" OR "south africans" OR "south sudanese" OR sudanese OR tanzanian OR tanzanians OR tanganyikan OR tanganyikans OR ugandan OR ugandans OR zambian OR zambians OR zimbabwean OR zimbabweans OR "sub-Saharan african" OR "subsaharan african" OR "East African" OR "Eastern African" OR "Central African" OR "southern african" OR "west african" OR "western african" OR "africa south of the sahara" OR "sub-Saharan africa" OR "subsaharan africa" OR "central african" OR "saharan" OR "southern african" OR "east african" OR "eastern african" OR "west african" OR "western african" ) | 1,300,821 |
| **Final** Combined | #1 AND #2 AND #3 | | ( INDEXTERMS ( "Anemia" OR "anemia, iron deficiency" OR "Hemoglobins" OR "Hematocrit" ) OR TITLE-ABS-KEY ( "Anemia during pregnancy" OR "Maternal anemia" OR "Anemia" OR "Maternal anaemia" OR "anaemia" OR "Hemoglobin" OR "maternal hemoglobin" OR "Haemoglobin" OR "Maternal Haemoglobin" OR "Hematocrit" OR "Maternal hematocrit" OR "Haematocrit" OR "Maternal haematocrit" OR "Maternal hemoglobin level" OR "Maternal hemoglobin concentration" ) ) AND ( INDEXTERMS ( "infant, low birth weight" OR "infant, extremely low birth weight" OR "infant, very low birth weight" OR "birth weight" ) OR TITLE-ABS-KEY ( "Low birth weight" OR "Adverse birth outcome" OR "Perinatal outcome" OR "Birth outcome" OR "birth weight" OR "low for gestational age" ) ) AND ( INDEXTERMS ( "Angola" OR "Benin" OR "Botswana" OR "Burkina Faso" OR "Burundi" OR "Cape Verde" OR "Cameroon" OR "Central African Republic" OR "Chad" OR "Comoros" OR "Democratic Republic Congo" OR "congo" OR "cote d` Ivoire" OR "Equatorial Guinea" OR "Eritrea" OR "Eswatini" OR "Ethiopia" OR "Gabon" OR "Gambia" OR "Ghana" OR "Guinea" OR "Guinea-Bissau" OR "Kenya" OR "Lesotho" OR "Liberia" OR "Madagascar" OR "Malawi" OR "Mali" OR "Mauritania" OR "Mauritius" OR "Mozambique" OR "Namibia" OR "Niger" OR "Nigeria" OR "Rwanda" OR "Sao Tome and Principe" OR "Senegal" OR "Seychelles" OR "Somalia" OR "South Africa" OR "South Sudan" OR "Sudan" OR "Tanzania" OR "Togo" OR "Uganda" OR "Zambia" OR "Zimbabwe" OR "Africa south of the Sahara" OR "Central Africa" ) OR TITLE-ABS-KEY ( angola OR benin OR "burkina faso" OR "burkina fasso" OR burundi OR urundi OR "cabo verde" OR "cape verde" OR cameroon OR cameron OR cameroun OR "central african republic" OR chad OR comoros OR "democratic republic of the congo" OR "democratic republic congo" OR congo OR zaire OR "cote d'ivoire" OR "cote d' ivoire" OR "cote divoire" OR "cote d ivoire" OR "ivory coast" OR "equatorial guinea" OR eritrea OR eswatini OR ethiopia OR gabon OR "gabonese republic" OR gambia OR ghana OR guinea OR "guinea bissau" OR kenya OR lesotho OR liberia OR madagascar OR malawi OR mali OR mauritania OR mauritius OR mozambique OR namibia OR niger OR nigeria OR rwanda OR ruanda OR "sao tome and principe" OR senegal OR seychelles OR "sierra leone" OR somalia OR "south africa" OR "south sudan" OR sudan OR tanzania OR tanganyika OR togo OR "togolese republic" OR uganda OR zambia OR zimbabwe OR "africa south of the sahara" OR "sub saharan africa" OR "subsaharan africa" OR "africa, central" OR "central africa" OR "africa, northern" OR "north africa" OR "northern africa" OR sahara OR "africa, southern" OR "southern africa" OR "africa, eastern" OR "east africa" OR "eastern africa" OR "africa, western" OR "west africa" OR "western africa" ) OR TITLE-ABS-KEY ( angolan OR beninese OR benineses OR botswana OR batswana OR burkinabe OR burkinese OR burundian OR burundians OR "cape verdean" OR "cape verdeans" OR "cabo verdean" OR "cabo verdeans" OR cameroonian OR cameroonians OR "central african" OR "central africans" OR chadian OR chadians OR comorian OR comorians OR congolese OR ivorian OR ivorians OR "equatorial guinean" OR "equatorial guineans" OR equatoguinean OR equatoguineans OR eritrean OR eritreans OR ethiopian OR ethiopians OR gabonese OR gabonaise OR gambian OR gambians OR ghanaian OR ghanaians OR guinean OR guineans OR "bissau guinean" OR "bissau guineans" OR kenyan OR kenyans OR lesothan OR lesothans OR lesothonian OR lesothonians OR liberian OR liberians OR madagascan OR madagascans OR malawian OR malawians OR malian OR malians OR mauritanian OR mauritanians OR mauritian OR mauritians OR mozambican OR mozambicans OR namibian OR namibians OR nigerien OR nigeriens OR nigerian OR nigerians OR rwandan OR rwandans OR rwandese OR ruandan OR ruandans OR ruandese OR "sao tomean" OR "sao tomeans" OR santomean OR santomeans OR senegalese OR seychellois OR seychelloise OR seychelloises OR "sierra leonean" OR "sierra leoneans" OR somali OR somalis OR "south african" OR "south africans" OR "south sudanese" OR sudanese OR tanzanian OR tanzanians OR tanganyikan OR tanganyikans OR ugandan OR ugandans OR zambian OR zambians OR zimbabwean OR zimbabweans OR "sub-Saharan african" OR "subsaharan african" OR "East African" OR "Eastern African" OR "Central African" OR "southern african" OR "west african" OR "western african" OR "africa south of the sahara" OR "sub-Saharan africa" OR "subsaharan africa" OR "central african" OR "saharan" OR "southern african" OR "east african" OR "eastern african" OR "west african" OR "western african" ) ) | 1,137 |
| Filters | limit to (English language and yr="2015 -Current" and article) | | | 1,021 |

| **Web of science data base** **Final Searching Date: June 12, 2024** | | | | |
| --- | --- | --- | --- | --- |
| **Concept** | **Line** | **Search term** | **Search strategy** | **Records** |
| Anemia | #1 | Anemia during pregnancy, Maternal anemia, anemia, Maternal anaemia, anaemia, Hemoglobin, maternal hemoglobin, Haemoglobin, Maternal Haemoglobin, hematocrit, Maternal hematocrit, Haematocrit,  Maternal haematocrit, Maternal hemoglobin level,  Maternal hemoglobin concentration | TS=("Anemia during pregnancy" OR "Maternal anemia" OR "Anemia" OR "Maternal anaemia" OR "anaemia" OR "Hemoglobin" OR "maternal hemoglobin" OR "Haemoglobin" OR "Maternal Haemoglobin" OR "Hematocrit" OR "Maternal hematocrit" OR "Haematocrit" OR "Maternal haematocrit" OR "Maternal hemoglobin level" OR "Maternal hemoglobin concentration" ) | 340,683 |
| Low birth weight | #2 | Low birth weight,  Adverse birth outcome, Perinatal outcome,  Birth outcome,  Birth weight,  Low for gestational age | TS=("Low birth weight" OR "Adverse birth outcome" OR "Perinatal outcome" OR "Birth outcome" OR "birth weight" OR "low for gestational age") | 102,389 |
| Sub-Saharan African Countries | #3 | Angola, Benin, Botswana,  Burkina Faso, Burundi, Cape Verde, Cameroon, Central African Republic, Chad, Comoros, Democratic Republic of the Congo, Cote D'ivoire, Equatorial Guinea,  Eritrea, Eswatini, Ethiopia, Gabon, Gambia, Ghana, Guinea, Guinea-Bissau, Kenya, Lesotho, Liberia, Madagascar, Malawi, Mali, Mauritania, Mauritius, Mozambique, Namibia, Niger, Nigeria, Rwanda, Sao Tome and Principe, Senegal, Seychelles, Sierra Leone, Somalia, South Africa, South Sudan, Sudan, Tanzania, Togo, Uganda, Zambia, Zimbabwe, africa south of the sahara, Sub-Saharan africa, subsaharan africa, central africa, north africa, northern africa, sahara, southern africa, east africa, eastern africa, West africa, western africa | TS=( angola OR benin OR "burkina faso" OR "burkina fasso" OR burundi OR urundi OR "cabo verde" OR "cape verde" OR cameroon OR cameron OR cameroun OR "central african republic" OR chad OR comoros OR "democratic republic of the congo" OR "democratic republic congo" OR congo OR zaire OR "cote d'ivoire" OR "cote d' ivoire" OR "cote divoire" OR "cote d ivoire" OR "ivory coast" OR "equatorial guinea" OR eritrea OR eswatini OR ethiopia OR gabon OR "gabonese republic" OR gambia OR ghana OR guinea OR "guinea bissau" OR kenya OR lesotho OR liberia OR madagascar OR malawi OR mali OR mauritania OR mauritius OR mozambique OR namibia OR niger OR nigeria OR rwanda OR ruanda OR "sao tome and principe" OR senegal OR seychelles OR "sierra leone" OR somalia OR "south africa" OR "south sudan" OR sudan OR tanzania OR tanganyika OR togo OR "togolese republic" OR uganda OR zambia OR zimbabwe OR "africa south of the sahara" OR "sub saharan africa" OR "subsaharan africa" OR "africa, central" OR "central africa" OR "africa, northern" OR "north africa" OR "northern africa" OR sahara OR "africa, southern" OR "southern africa" OR "africa, eastern" OR "east africa" OR "eastern africa" OR "africa, western" OR "west africa" OR "western africa" OR angolan OR beninese OR benineses OR botswana OR batswana OR burkinabe OR burkinese OR burundian OR burundians OR "cape verdean" OR "cape verdeans" OR "cabo verdean" OR "cabo verdeans" OR cameroonian OR cameroonians OR "central african" OR "central africans" OR chadian OR chadians OR comorian OR comorians OR congolese OR ivorian OR ivorians OR "equatorial guinean" OR "equatorial guineans" OR equatoguinean OR equatoguineans OR eritrean OR eritreans OR ethiopian OR ethiopians OR gabonese OR gabonaise OR gambian OR gambians OR ghanaian OR ghanaians OR guinean OR guineans OR "bissau guinean" OR "bissau guineans" OR kenyan OR kenyans OR lesothan OR lesothans OR lesothonian OR lesothonians OR liberian OR liberians OR madagascan OR madagascans OR malawian OR malawians OR malian OR malians OR mauritanian OR mauritanians OR mauritian OR mauritians OR mozambican OR mozambicans OR namibian OR namibians OR nigerien OR nigeriens OR nigerian OR nigerians OR rwandan OR rwandans OR rwandese OR ruandan OR ruandans OR ruandese OR "sao tomean" OR "sao tomeans" OR santomean OR santomeans OR senegalese OR seychellois OR seychelloise OR seychelloises OR "sierra leonean" OR "sierra leoneans" OR somali OR somalis OR "south african" OR "south africans" OR "south sudanese" OR sudanese OR tanzanian OR tanzanians OR tanganyikan OR tanganyikans OR ugandan OR ugandans OR zambian OR zambians OR zimbabwean OR zimbabweans OR "sub-Saharan african" OR "subsaharan african" OR "East African" OR "Eastern African" OR "Central African" OR "southern african" OR "west african" OR "western african" OR "africa south of the sahara" OR "sub-Saharan africa" OR "subsaharan africa" OR "central african" OR "saharan" OR "southern african" OR "east african" OR "eastern african" OR "west african" OR "western african" ) | 867,111 |
| **Final** Combined |  | (TS=("Anemia during pregnancy" OR "Maternal anemia" OR "Anemia" OR "Maternal anaemia" OR "anaemia" OR "Hemoglobin" OR "maternal hemoglobin" OR "Haemoglobin" OR "Maternal Haemoglobin" OR "Hematocrit" OR "Maternal hematocrit" OR "Haematocrit" OR "Maternal haematocrit" OR "Maternal hemoglobin level" OR "Maternal hemoglobin concentration" )) AND (TS=("Low birth weight" OR "Adverse birth outcome" OR "Perinatal outcome" OR "Birth outcome" OR "birth weight" OR "low for gestational age")) AND (TS=( angola OR benin OR "burkina faso" OR "burkina fasso" OR burundi OR urundi OR "cabo verde" OR "cape verde" OR cameroon OR cameron OR cameroun OR "central african republic" OR chad OR comoros OR "democratic republic of the congo" OR "democratic republic congo" OR congo OR zaire OR "cote d'ivoire" OR "cote d' ivoire" OR "cote divoire" OR "cote d ivoire" OR "ivory coast" OR "equatorial guinea" OR eritrea OR eswatini OR ethiopia OR gabon OR "gabonese republic" OR gambia OR ghana OR guinea OR "guinea bissau" OR kenya OR lesotho OR liberia OR madagascar OR malawi OR mali OR mauritania OR mauritius OR mozambique OR namibia OR niger OR nigeria OR rwanda OR ruanda OR "sao tome and principe" OR senegal OR seychelles OR "sierra leone" OR somalia OR "south africa" OR "south sudan" OR sudan OR tanzania OR tanganyika OR togo OR "togolese republic" OR uganda OR zambia OR zimbabwe OR "africa south of the sahara" OR "sub saharan africa" OR "subsaharan africa" OR "africa, central" OR "central africa" OR "africa, northern" OR "north africa" OR "northern africa" OR sahara OR "africa, southern" OR "southern africa" OR "africa, eastern" OR "east africa" OR "eastern africa" OR "africa, western" OR "west africa" OR "western africa" OR angolan OR beninese OR benineses OR botswana OR batswana OR burkinabe OR burkinese OR burundian OR burundians OR "cape verdean" OR "cape verdeans" OR "cabo verdean" OR "cabo verdeans" OR cameroonian OR cameroonians OR "central african" OR "central africans" OR chadian OR chadians OR comorian OR comorians OR congolese OR ivorian OR ivorians OR "equatorial guinean" OR "equatorial guineans" OR equatoguinean OR equatoguineans OR eritrean OR eritreans OR ethiopian OR ethiopians OR gabonese OR gabonaise OR gambian OR gambians OR ghanaian OR ghanaians OR guinean OR guineans OR "bissau guinean" OR "bissau guineans" OR kenyan OR kenyans OR lesothan OR lesothans OR lesothonian OR lesothonians OR liberian OR liberians OR madagascan OR madagascans OR malawian OR malawians OR malian OR malians OR mauritanian OR mauritanians OR mauritian OR mauritians OR mozambican OR mozambicans OR namibian OR namibians OR nigerien OR nigeriens OR nigerian OR nigerians OR rwandan OR rwandans OR rwandese OR ruandan OR ruandans OR ruandese OR "sao tomean" OR "sao tomeans" OR santomean OR santomeans OR senegalese OR seychellois OR seychelloise OR seychelloises OR "sierra leonean" OR "sierra leoneans" OR somali OR somalis OR "south african" OR "south africans" OR "south sudanese" OR sudanese OR tanzanian OR tanzanians OR tanganyikan OR tanganyikans OR ugandan OR ugandans OR zambian OR zambians OR zimbabwean OR zimbabweans OR "sub-Saharan african" OR "subsaharan african" OR "East African" OR "Eastern African" OR "Central African" OR "southern african" OR "west african" OR "western african" OR "africa south of the sahara" OR "sub-Saharan africa" OR "subsaharan africa" OR "central african" OR "saharan" OR "southern african" OR "east african" OR "eastern african" OR "west african" OR "western african" )) | | **679** |
| Filters | Limit publication date from Jan 2015 to June 2024, and Article Document type and English language | | | 336 |
