# Supplementary material for: Effects of maternal anemia on low-birth-weight in Sub-Sahara African countries: Systematic review and meta-analysis
Source: PLoS One. 2025 Jun 25;20(6):e0325450. doi: 10.1371/journal.pone.0325450 (PMC12192055; doi:10.1371/journal.pone.0325450)
Supplement: S2 Table — (DOCX) [file pone.0325450.s002.docx]

**S 2 Table: All data extracted from each study for the reported systematic review and meta-analysis**

| Author year | Countries | Study Design | Population | Sample size | Eligibility criteria | Adjusted Odd ratio | Name of data Extractor | Date of data extractor |
| --- | --- | --- | --- | --- | --- | --- | --- | --- |
| Engidaw et al. (2022) | Ethiopia | Cross sectional | Infants | 211 | Eligible | 3.51 | Abebe Gedefaw | June 26, 2024 |
| Seid et al. (2022) | Ethiopia | Case control | Infants | 252 | Eligible | 5.2 | Nigus Kabtu | June 26, 2024 |
| Elmugabil et al. (2023) | Sudan | Cross sectional | Infants | 253 | Eligible | 4.7 | Darik Temesgen | June 27, 2024 |
| Ahmed et al. (2018) | Ethiopia | Case control | Infants | 279 | Eligible | 3.54 | Darik Temesgen | June 26, 2024 |
| Girma et al. (2019) | Ethiopia | Case control | Infants | 279 | Eligible | 3.54 | Abebe Gedefaw | June 28, 2024 |
| Mekie and Taklual (2019) | Ethiopia | Cross sectional | Women | 282 | Eligible | 9.82 | Nigus Kabtu | June 27, 2024 |
| Gebrehawerya et al. (2018) | Ethiopia | Case control | Women | 287 | Eligible | 3.91 | Darik Temesgen | June 30, 2024 |
| Mingude et al. (2020) | Ethiopia | Case control | Women | 300 | Eligible | 4.4 | Nigus Kabtu | June 27, 2024 |
| Lake and Fite (2019) | Ethiopia | Cross sectional | Infants | 304 | Eligible | 3.808 | Nigus Kabtu | June 29, 2024 |
| Aboye et al. (2018) | Ethiopia | Cross sectional | Women | 308 | Eligible | 14.5 | Abebe Gedefaw | June 30, 2024 |
| Tadesse et al. (2023) | Ethiopia | Cross sectional | Infants | 337 | Eligible | 4.34 | Darik Temesgen | June 28, 2024 |
| Abera et al. (2019) | Ethiopia | Cross sectional | Women | 358 | Eligible | 3.42 | Nigus Kabtu | June 29, 2024 |
| Adam et al. (2019) | Ghana | Case control | Women | 360 | Eligible | 3.14 | Abebe Gedefaw | June 30, 2024 |
| Hailu et al. (2021) | Ethiopia | Cross sectional | Women | 363 | Eligible | 4.99 | Darik Temesgen | June 28, 2024 |
| Kumlachew et al. (2018) | Ethiopia | Cross sectional | Women | 375 | Eligible | 2.69 | Nigus Kabtu | June 27, 2024 |
| Muluneh et al. (2023) | Ethiopia | Cross sectional | Infants | 422 | Eligible | 5.87 | Abebe Gedefaw | June 28, 2024 |
| Kargbo et al. (2021) | Sierra Leone | Case control | Women | 438 | Eligible | 3.8 | Nigus Kabtu | June 29, 2024 |
| Deriba and jemal (2021 | Ethiopia | Case control | Women | 555 | Eligible | 2.34 | Abebe Gedefaw | June 29, 2024 |
| Oladeinde et al 2015 | Nigeria | Cross sectional | Women | 780 | Eligible | 2.79 | Abebe Gedefaw | June 30, 2024 |
| Biracyaza et al. (2021) | Rwanda | Cross sectional | Women | 7381 | Eligible | 3.5 | Darik Temesgen | June 28, 2024 |
| Mitao et al. (2016) | Tanzania | Retrospective Cohort | Women | 37799 | Eligible | 1.58 | Darik Temesgen | June 27, 2024 |
