# Supplementary material for: Effects of maternal anemia on low-birth-weight in Sub-Sahara African countries: Systematic review and meta-analysis
Source: PLoS One. 2025 Jun 25;20(6):e0325450. doi: 10.1371/journal.pone.0325450 (PMC12192055; doi:10.1371/journal.pone.0325450)
Supplement: S3 Table — (DOCX) [file pone.0325450.s003.docx]

**S 3 Table: Risk of bias and quality/certainty assessments for included studies.**

**Table S3 A: Risk of bias and quality/certainty assessments for cross sectional studies.**

| **Code** | **Author ­_Year** | **Q1** | **Q2** | **Q3** | **Q4** | **Q5** | **Q6** | **Q7** | **Q8** | **Total Score** | **Quality of Study** |
| --- | --- | --- | --- | --- | --- | --- | --- | --- | --- | --- | --- |
| 1 | Engidaw et al. (2022) | 1 | 1 | 1 | 1 | 1 | 1 | 1 | 1 | 8 | **Good** |
| 2 | Elmugabil et al. (2023) | 1 | 1 | 1 | 1 | 1 | 0 | 1 | 1 | 7 | **Good** |
| 3 | Mekie and Taklual (2019) | 1 | 1 | 1 | 1 | 1 | 1 | 1 | 1 | 8 | **Good** |
| 4 | Lake and Fite (2019) | 1 | 1 | 1 | 1 | 1 | 1 | 1 | 1 | 8 | **Good** |
| 5 | Aboye et al. (2018) | 0 | 1 | 1 | 1 | 1 | 1 | 1 | 1 | 7 | **Good** |
| 6 | Tadesse et al. (2023) | 1 | 1 | 1 | 1 | 1 | 1 | 1 | 1 | 8 | **Good** |
| 7 | Abera et al. (2019) | 1 | 1 | 1 | 1 | 1 | 1 | 1 | 1 | 8 | **Good** |
| 8 | Hailu et al. (2021) | 1 | 1 | 0 | 1 | 1 | 1 | 1 | 1 | 7 | **Good** |
| 9 | Kumlachew et al. (2018) | 0 | 1 | 1 | 1 | 1 | 1 | 1 | 1 | 7 | **Good** |
| 10 | Muluneh et al. (2023 | 1 | 1 | 1 | 1 | 1 | 1 | 1 | 1 | 8 | **Good** |
| 11 | Oladeinde et al 2015 | 1 | 1 | 0 | 1 | 0 | 0 | 1 | 1 | 5 | **Moderate** |
| 12 | Biracyaza et al. (2021) | 0 | 1 | 1 | 1 | 1 | 1 | 1 | 1 | 7 | **Good** |

**Note:**

**1= yes**

**0= no/unclear/not applicable**

- **Good (6-10 score) or 80–100% items scored yes**
- **Moderate (4-6 score) or 50–80% items scored yes**
- **Low (1- 4) or 20–50% items scored yes**

**Quality assessment questions customized from JBI critical appraisal checklist for analytical cross-sectional studies**

Q1: Were the criteria for inclusion in the sample clearly defined?

Q2: Were the study subjects and the setting described in detail?

Q3: Was the exposure measured in a valid and reliable way?

Q4: Were objective, standard criteria used for measurement of the condition?

Q5: Were confounding factors identified? Q6: Were strategies to deal with confounding factors stated?

Q7: Were the outcomes measured in a valid and reliable way?

| **Code** | **Author ­_Year** | **Q1** | **Q2** | **Q3** | **Q4** | **Q5** | **Q6** | **Q7** | **Q8** | **Q9** | **Q10** | **Total Score** | **Quality of Study** |
| --- | --- | --- | --- | --- | --- | --- | --- | --- | --- | --- | --- | --- | --- |
| 1 | Seid et al. (2022) | 1 | 1 | 1 | 1 | 1 | 1 | 1 | 1 | 1 | 1 | 10 | **Good** |
| 2 | Ahmed et al. (2018) | 1 | 1 | 1 | 1 | 1 | 1 | 1 | 1 | 1 | 1 | 10 | **Good** |
| 3 | Girma et al. (2019) | 1 | 1 | 1 | 1 | 1 | 1 | 1 | 1 | 1 | 1 | 10 | **Good** |
| 4 | Gebrehawerya et al. (2018) | 1 | 1 | 1 | 1 | 1 | 1 | 1 | 1 | 1 | 1 | 10 | **Good** |
| 5 | Mingude et al. (2020) | 1 | 1 | 1 | 1 | 1 | 1 | 1 | 1 | 1 | 1 | 10 | **Good** |
| 6 | Adam et al. (2019) | 1 | 1 | 1 | 1 | 1 | 1 | 1 | 1 | 0 | 1 | 9 | **Good** |
| 7 | Kargbo et al. (2021) | 1 | 1 | 1 | 1 | 1 | 1 | 1 | 1 | 1 | 1 | 10 | **Good** |
| 8 | Deriba and jemal (2021) | 1 | 1 | 1 | 1 | 1 | 1 | 1 | 1 | 1 | 1 | 10 | **Good** |

Q8: Was appropriate statistical analysis used?

**Table S3B: Risk of bias and quality/certainty assessments for Case control studies.**

**Note:**

**1= yes**

**0= no/unclear/not applicable**

- **Good (8-10 score) or 80–100% items scored yes**
- **Moderate (5-8 score) or 50–80% items scored yes**
- **Low (1- 5) or 20–50% items scored yes**

**Quality assessment questions customized from JBI critical appraisal checklist for analytical Case control studies**

Q1: Were the groups comparable other than the presence of disease in cases or the absence of disease in controls?

Q2: Were cases and controls matched appropriately?

Q3: Were the same criteria used for identification of cases and controls?

Q4: Was exposure measured in a standard, valid and reliable way?

Q5. Was exposure measured in the same way for cases and controls?

Q6: Were confounding factors identified?

Q7: Were strategies to deal with confounding factors stated?

Q8: Were outcomes assessed in a standard, valid and reliable way for cases and controls?

Q9: Was the exposure period of interest long enough to be meaningful?

Q10: Was appropriate statistical analysis used?

**Table S3 C: Risk of bias and quality/certainty assessments for cohort studies.**

| **Code** | **Author ­_Year** | **Q1** | **Q2** | **Q3** | **Q4** | **Q5** | **Q6** | **Q7** | **Q8** | **Q9** | **Q10** | **Q11** | **Total Score** | **Quality of Study** |
| --- | --- | --- | --- | --- | --- | --- | --- | --- | --- | --- | --- | --- | --- | --- |
| 1 | Mitao et al. (2016) | 1 | 1 | 1 | 1 | 0 | 1 | 0 | 1 | 1 | 0 | 1 | 8 | **Moderate** |

**Note:**

**1= yes**

**0= no/unclear/not applicable**

- **Good (8.8-10 score) or 80–100% items scored yes**
- **Moderate (5.5-8.8 score) or 50–80% items scored yes**
- **Low (1- 5.5) or 20–50% items scored yes**

**Quality assessment questions customized from JBI critical appraisal checklist for analytical Cohort studies**

Q1: Were the two groups similar and recruited from the same population?

Q2: Were the exposures measured similarly to assign people to both exposed and unexposed groups?

Q3: Was the exposure measured in a valid and reliable way?

Q4: Were confounding factors identified?

Q5: Were strategies to deal with confounding factors stated?

Q6: Were the groups/participants free of the outcome at the start of the study (or at the moment of exposure)?

Q7: Were the outcomes measured in a valid and reliable way?

Q8: Was the follow up time reported and sufficient to be long enough for outcomes to occur?

Q9: Was follow up complete, and if not, were the reasons to loss to follow up described and explored?

Q10: Were strategies to address incomplete follow up utilized?

Q11: Was appropriate statistical analysis used?
