# Supplementary material for: Effects of maternal anemia on low-birth-weight in Sub-Sahara African countries: Systematic review and meta-analysis
Source: PLoS One. 2025 Jun 25;20(6):e0325450. doi: 10.1371/journal.pone.0325450 (PMC12192055; doi:10.1371/journal.pone.0325450)
Supplement: S2 File — (PDF) [file pone.0325450.s005.pdf]

S2 File: Joanna Briggs Institute (JBI) checklist to assess the methodologic quality of all studies.

JBI critical appraisal checklist for analytical cross-sectional studies

Reviewer______________________________________

Date______________________________

Author____Year_________Record Number_________

	Yes	No	Unclear	Not applicable
1. Were the criteria for inclusion in the sample clearly defined?	☐	☐	☐	☐
2. Were the study subjects and the setting described in detail?	☐	☐	☐	☐
3. Was the exposure measured in a valid and reliable way?	☐	☐	☐	☐
4. Were objective, standard criteria used for measurement of the condition?	☐	☐	☐	☐
5. Were confounding factors identified?	☐	☐	☐	☐
6. Were strategies to deal with confounding factors stated?	☐	☐	☐	☐
7. Were the outcomes measured in a valid and reliable way?	☐	☐	☐	☐
8. Was appropriate statistical analysis used?	☐	☐	☐	☐

Overall appraisal: Include ☐ Exclude ☐ Seek further info ☐

JBI critical appraisal checklist for case control studies

Reviewer______________________________________

Date______________________________

Author_____________________________________Year_________Record Number_________

	Yes	No	Unclear	Not applicable
1. Were the groups comparable other than the presence of disease in cases or the absence of disease in controls?	☐	☐	☐	☐
2. Were cases and controls matched appropriately?	☐	☐	☐	☐
3. Were the same criteria used for identification of cases and controls?	☐	☐	☐	☐
4. Was exposure measured in a standard, valid and reliable way?	☐	☐	☐	☐
5. Was exposure measured in the same way for cases and controls?	☐	☐	☐	☐
6. Were confounding factors identified?	☐	☐	☐	☐
7. Were strategies to deal with confounding factors stated?	☐	☐	☐	☐
8. Were outcomes assessed in a standard, valid and reliable way for cases and controls?	☐	☐	☐	☐
9. Was the exposure period of interest long enough to be meaningful?	☐	☐	☐	☐
10. Was appropriate statistical analysis used?	☐	☐	☐	☐

Overall appraisal: Include ☐ Exclude ☐ Seek further info ☐

JBI critical appraisal checklist for cohort studies

Reviewer____

Date__________________________________

Author____Year__________Record Number__________

	Yes	No	Unclear	Not applicable
1. Were the two groups similar and recruited from the same population?	☐	☐	☐	☐
2. Were the exposures measured similarly to assign people to both exposed and unexposed groups?	☐	☐	☐	☐
3. Was the exposure measured in a valid and reliable way?	☐	☐	☐	☐
4. Were confounding factors identified?	☐	☐	☐	☐
5. Were strategies to deal with confounding factors stated?	☐	☐	☐	☐
6. Were the groups/participants free of the outcome at the start of the study (or at the moment of exposure)?	☐	☐	☐	☐
7. Were the outcomes measured in a valid and reliable way?	☐	☐	☐	☐
8. Was the follow up time reported and sufficient to be long enough for outcomes to occur?	☐	☐	☐	☐

9. Was follow up complete, and if not, were the reasons to loss to follow up described and explored? ☐ ☐ ☐ ☐

10. Were strategies to address incomplete follow up utilized? ☐ ☐ ☐ ☐

11. Was appropriate statistical analysis used? ☐ ☐ ☐ ☐

Overall appraisal: Include ☐ Exclude ☐ Seek further info ☐

Comments (Including reason for exclusion)

____

____

____

______
