# Supplementary material for: Effects of maternal anemia on low-birth-weight in Sub-Sahara African countries: Systematic review and meta-analysis
Source: PLoS One. 2025 Jun 25;20(6):e0325450. doi: 10.1371/journal.pone.0325450 (PMC12192055; doi:10.1371/journal.pone.0325450)
Supplement: Attachment — Submitted filename: Response to Reviewers.docx [file pone.0325450.s008.docx]

**Response to academic editor and reviewer comment**

Thank you for your insightful and supportive feedback. We have addressed all concerns raised by the editor and reviewer, and I am happy to clarify any remaining issues in the future.

| S.no | **Editor comment** | **Authors response** |
| --- | --- | --- |
| 1. | Please ensure that your manuscript meets PLOS ONE's style requirements, including those for file naming. | Accepted and corrected according to the guideline. |
| 2 | We noticed you have some minor occurrence of overlapping text with the following previous publication(s), which needs to be addressed: | Accepted and corrected: We tried to paraphrase again. |
| 3 | A numbered table of all studies identified in the literature search, including those that were excluded from the analyses.  For every excluded study, the table should list the reason(s) for exclusion. | Accepted: We add **S 2 Table** as a supporting information that include all studies including the exclude one and reason for exclusion. |
|  | A table of all data extracted from the primary research sources for the systematic review and/or meta-analysis. The table must include the following information for each study:  Name of data extractors and date of data extraction  Confirmation that the study was eligible to be included in the review.  All data extracted from each study for the reported systematic review and/or meta-analysis that would be needed to replicate your analyses. | Accepted: We add **S 4 Table** as a supporting information have all extracted data, confirmation for eligibility, name of data extractor, and data of data extraction. |
| 4 | We note that you have indicated that there are restrictions to data sharing for this study. | Corrected: The datasets analyzed during the current study are available in **S5 file** and attached as a supporting information. |
| 5 | In the online submission form, you indicated that the datasets analyzed during the current study are available from the corresponding author on reasonable request. |  |
| 6 | Please include captions for your Supporting Information files at the end of your manuscript, and update any in-text citations to match accordingly. | Accepted and corrected: We put the citations of all supporting information at the end of the manuscript below the reference and update the citation. |
|  | **Reviewer Comments Author Responses** | |
| 1. | Reviewer #1: question 4 comment: Requires careful editing for proper English grammar | Accepted: We tried to edit each sentence and paragraph throughout the document. |
| 2. | The sizable impact of low birth weight in neonates and its link to mortality and morbidity has long been known.  Many published papers, but certainly not all, have reported associated between iron deficiency anemia in pregnancy and low birth weight. It is difficult for a reader to determine if the meta-analysis and systematic review was focused on anemia or on iron deficiency anemia. | Accepted and corrected: Thank you for your review. A paragraph describing anemia burden and consequences has been added (P3 L48-54). This study focuses on anemia generally and does not assess iron deficiency anemia independently. |
| 3. | The authors correctly point to the importance of adequate nutrition as a preventive measure to reduce low birth weight.  Rates of iron indices in pregnancy vary greatly among African countries. These rates are impacted by factors such as iron in groundwater, a deficiency in related micronutrients, the presence of helminths, HIV/AIDS, body weight, age, prior pregnancy history, and perhaps most importantly the hemoglobin level (and ferritin) present when a woman enters pregnancy. | I appreciate your concern and agree with your suggestions. The manuscript highlights nutritional intervention packages as a preventive measure, alongside other cause-specific interventions like infection prevention and management. |
| 4. | Of the 21 studies reviewed, it is important to note that none were randomized, placebo-controlled trials. While the association of anemia to lower birth weight in anemic women during pregnancy appears to be confirmed, it is difficult to extrapolate the importance of these findings on actionable approaches to care.  While the standards for a systematic review and metanalysis are well maintained, the reported findings may not be generalizable. | Thank you for your concern and pertinent questions. This study included cohort, case-control, and cross-sectional designs, but excluded experimental designs. While experimental studies offer high-quality evidence, their interventional nature, where an effect follows a specific action, makes them difficult to integrate with observational study designs. Therefore, the best approach was to exclude them or conduct a separate with only similar experimental studies. |
